# Physiological IRE-1-XBP-1 and PEK-1 Signaling in *Caenorhabditis elegans* Larval Development and Immunity

**DOI:** 10.1371/journal.pgen.1002391

**Published:** 2011-11-17

**Authors:** Claire E. Richardson, Stephanie Kinkel, Dennis H. Kim

**Affiliations:** Department of Biology, Massachusetts Institute of Technology, Cambridge, Massachusetts, United States of America; University of California San Francisco, United States of America

## Abstract

Endoplasmic reticulum (ER) stress activates the Unfolded Protein Response, a compensatory signaling response that is mediated by the IRE-1, PERK/PEK-1, and ATF-6 pathways in metazoans. Genetic studies have implicated roles for UPR signaling in animal development and disease, but the function of the UPR under physiological conditions, in the absence of chemical agents administered to induce ER stress, is not well understood. Here, we show that in *Caenorhabditis elegans* XBP-1 deficiency results in constitutive ER stress, reflected by increased basal levels of IRE-1 and PEK-1 activity under physiological conditions. We define a dynamic, temperature-dependent requirement for XBP-1 and PEK-1 activities that increases with immune activation and at elevated physiological temperatures in *C. elegans*. Our data suggest that the negative feedback loops involving the activation of IRE-1-XBP-1 and PEK-1 pathways serve essential roles, not only at the extremes of ER stress, but also in the maintenance of ER homeostasis under physiological conditions.

## Author Summary

Proteins destined for secretion outside of eukaryotic cells are trafficked through the endoplasmic reticulum (ER). Protein folding in the ER involves the activity of chaperones, as well as catalysis of post-translational modifications such as disulfide bond formation and glycosylation. When the folding capacity of the ER is exceeded, the resulting accumulation of misfolded proteins activates the Unfolded Protein Response (UPR), a conserved signaling response that functions to restore protein folding homeostasis in the ER. Genetic studies have established that the UPR is required for the development of specific cell types in mammals, such as antibody-secreting plasma cells, and recent studies implicate a critical role for UPR signaling in the pathogenesis of metabolic and inflammatory diseases. In this paper we show that innate immunity and elevated physiological temperatures each necessitate UPR activity for *C. elegans* survival. Furthermore, we show that, under physiological conditions of larval development, basal activity of the UPR is required for the maintenance of ER homeostasis. Our data support the idea not only that the UPR functions as a “stress response” pathway, protecting against the extremes of unfolded protein accumulation, but also that the UPR plays a more general role in animal physiology and development.

## Introduction

The accumulation of misfolded proteins in the endoplasmic reticulum (ER), also known as ER stress, activates the Unfolded Protein Response (UPR), which upregulates the synthesis of chaperones such as BiP and components of ER-associated degradation (ERAD), promotes ER expansion, and attenuates translation [1]–[3]. The UPR is conserved from yeast to humans and in metazoans is comprised of three branches, mediated by the transmembrane ER luminal sensors IRE-1, PERK/PEK-1, and ATF-6 [1]–[3]. In response to ER stress, IRE-1 oligomerizes, activating an endoribonuclease domain that splices the mRNA of *xbp-1* to enable the generation of the activated form of the XBP-1 transcription factor [4]–[7]. PERK phosphorylates the translation initiation factor eIF-2α, causing global translational attenuation that diminishes the secretory load to the ER [8]. In addition, phosphorylation of eIF-2α selectively increases the translation of ATF4, a transcription factor that regulates stress responses [9]. ATF-6 undergoes proteolysis, releasing the cytosolic domain of ATF-6, which functions as a transcription factor that translocates to the nucleus and activates transcription of UPR genes [10].

Genetic studies suggest essential roles for UPR signaling in animal development. In mice, genetic studies focused on either the IRE-1-XBP-1 or the PERK pathway have shown that each functions in the development of specialized cell types, including plasma cells, pancreatic ß-cells, hepatocytes, and intestinal epithelial cells [3], [11]–[15]. In *Caenorhabditis elegans*, mutants deficient in any one of the three branches of the UPR are viable, but combining a deficiency in the IRE-1-XBP-1 pathway with loss-of-function mutations in either the ATF-6 or PEK-1 branch has been reported to result in larval lethality [6], [16]. These studies suggest that the UPR is required for animal development, but the specific essential role has not been defined. For example, UPR signaling may be required for a particular stage of development, or alternatively, constitutive UPR activity may be required. The experimental analysis of UPR signaling both in yeast and in mammalian cells has been greatly facilitated by the use of chemical agents that induce ER stress, such as the N-linked glycosylation inhibitor tunicamycin, the calcium pump inhibitor thapsigargin, and the reducing agent dithiothreitol (DTT). However, the activation of the UPR under physiological conditions is less well understood [17]. Constitutive IRE-1 activity has been observed in diverse types of mammalian cells, particularly with high secretory activity or in the setting of increased inflammatory signaling [11], [14], [15], [18]. These studies suggest critical roles for IRE-1-XBP-1 signaling in physiology and development, some of which have been proposed to be independent of its role in maintaining protein folding homeostasis in the ER [11], [19], [20].

Recently, we showed that XBP-1 is required for *C. elegans* larval development on pathogenic *Pseudomonas aeruginosa*, conferring protection to the *C. elegans* host against the ER stress caused by its own secretory innate immune response to infection [21]. Our study established that the innate immune response to microbial pathogens represents a physiologically relevant source of ER stress that necessitates XBP-1 function.

We sought to better understand the consequences of UPR deficiency under physiological conditions during *C. elegans* larval development. We describe our studies which suggest that even in the absence of ER stress induced by exogenously administered chemical agents, the IRE-1-XBP-1 pathway, in concert with the PEK-1 pathway, functions in a homeostatic loop that is under constitutive activation during *C. elegans* larval development. Our data implicate an essential role for the UPR in ER homeostasis, not only in the response to toxin-induced ER stress, but also under basal physiological conditions.

## Results

### Increased Constitutive IRE-1 Activity in *C. elegans xbp-1* Mutants

The detection of IRE-1 activity provides a sensitive and responsive measure of ER stress. Most methods used to measure IRE-1 activity require functional IRE-1-XBP-1 output, relying on either detection of the activated spliced form of the *xbp-1* mRNA or the transcriptional activity of the resulting XBP-1 protein. In order to follow IRE-1 activity in the absence of a functional XBP-1 protein, we utilized the *C. elegans xbp-1(zc12)* mutant, which has a C→T mutation that results in an early premature stop codon [4]. We reasoned that we could detect IRE-1-mediated splicing of *xbp-1(zc12)* mRNA by quantitative RT-PCR (qPCR), as we have done previously for wild type *xbp-1* mRNA, as a measure of IRE-1 activity [21].

**Figure 1:**
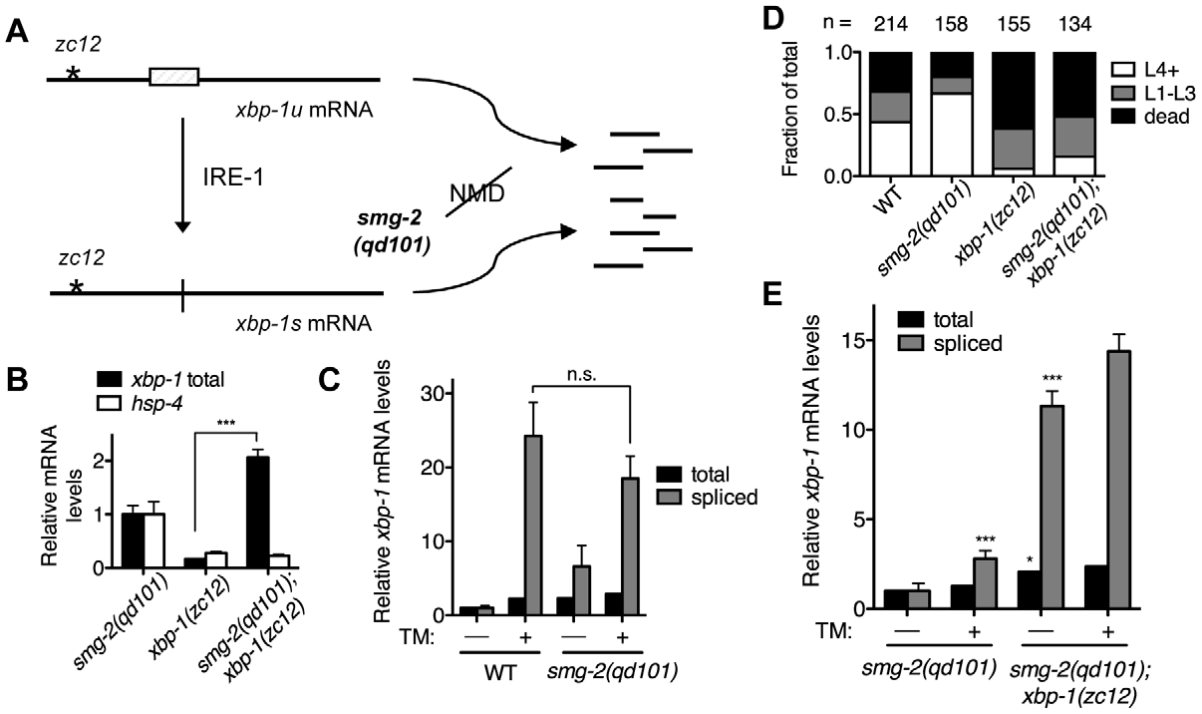
XBP-1 deficiency results in a dramatic increase in IRE-1 activity. (A) Detection of *xbp-1* mRNA splicing by IRE-1 in the *C. elegans xbp-1(zc12)* mutant. Schematic of the unspliced and spliced *xbp-1* mRNA, noting the position of the premature termination codon present in the *xbp-1(zc12)* allele. The *smg-2(qd101)* mutation results in inactivation of the NMD pathway, stabilizing the *xbp-1(zc12)* mRNA for detection. (B) Quantitative real-time PCR measurements of mRNA levels in the *xbp-1(zc12)* and *smg-2(qd101);xbp-1(zc12)* mutants relative to the *smg-2(qd101)* mutant synchronized in the L3 stage. (C) Quantitative real-time PCR measurements of levels of total and spliced *xbp-1* mRNA in the *smg-2(qd101)* strain relative to WT grown to the L3 stage and then shifted to plates with or without tunicamycin (5 µg/mL) for 4 h. (D) Larval development and survival assay showing the proportion of animals of each of the indicated strains that reach the indicated stage after 4 d of development from eggs laid on plates containing tunicamycin (2.5 µg/mL) at 16°C. (E) Quantitative real-time PCR measurements of levels of total and spliced *xbp-1* mRNA in the *smg-2(qd101)*; *xbp-1(zc12)* strain relative to the *smg-2(qd101)* strain treated as in C. (In B, C, and E, values represent fold change ± s.e.m., n = 3 independent experiments, *P<0.05, ***P<0.001, two-way ANOVA with Bonferroni post test).

We anticipated, however, that the *xbp-1(zc12)* mRNA might be degraded by nonsense-mediated decay (NMD) [22], which would reduce the abundance of *xbp-1(zc12)* mRNA (Figure 1A). Thus, we constructed a strain carrying *xbp-1(zc12)* and a null allele of *smg-2*, the *C. elegans* homolog of the NMD component Upf1 [23]. Indeed, we observed that the level of *xbp-1* mRNA in the *xbp-1(zc12)* mutant was markedly diminished compared with the level of *xbp-1(zc12)* mRNA in the *smg-2(qd101)*; *xbp-1(zc12)* mutant (Figure 1B). These data confirmed that *xbp-1(zc12)* mRNA is a substrate for the NMD pathway, but that inhibition of NMD permits detection of *xbp-1(zc12)* mRNA. As expected from the predicted truncated protein product made from translation of the *xbp-1(zc12)* mRNA (Figure 1A), loss of NMD had no effect on the null phenotype of the *xbp-1(zc12)* allele, as assessed by the effect of the *smg-2(qd101)* mutation on expression of an *xbp-1*-regulated gene, the *C. elegans* BiP homolog *hsp-4* (Figure 1B). We next examined the level of WT *xbp-1* mRNA in the *smg-2(qd101)* mutant, and we observed that NMD inhibition increased the level of *xbp-1* mRNA 2-fold relative to WT *C. elegans* (Figure 1C), which suggests that the NMD complex may function to decrease the level of WT *xbp-1* mRNA. This observation is consistent with a prior report suggesting that stress-induced genes may be NMD targets [24], although we hypothesize that the relatively early termination codon present in the *xbp-1* mRNA prior to IRE-1-mediated splicing may also contribute to recognition and degradation by the NMD pathway. Consistent with this explanation, after exposing both the WT and *smg-2(qd101)* strains to tunicamycin for 4 h, the level of IRE-1-spliced *xbp-1* mRNA was similar between the two strains (Figure 1C). Furthermore, the loss of NMD did not increase the lethality of either the WT strain or *xbp-1(zc12)* mutant when grown in the presence of tunicamycin (Figure 1D).

Comparing levels of IRE-1 activity between *smg-2(qd101)* and *smg-2(qd101)*; *xbp-1(zc12)* animals, we observed a dramatic elevation in the level of spliced *xbp-1* mRNA in the *smg-2(qd101)*; *xbp-1(zc12)* strain (Figure 1E). To provide a measure for comparison, the basal elevation of spliced *xbp-1* mRNA in the *smg-2(qd101)*; *xbp-1(zc12)* mutant far exceeded the level of spliced *xbp-1* mRNA in the *smg-2(qd101)* mutant even after administration of tunicamycin. Treating the *smg-2(qd101)*; *xbp-1(zc12)* strain with tunicamycin resulted in only a minor additional increase in spliced *xbp-1* mRNA compared with the magnitude of elevation in spliced *xbp-1* in that strain under basal conditions (Figure 1E). These data show that XBP-1 deficiency results in a dramatic increase in IRE-1 activity, even in the absence of exogenously administered agents such as tunicamycin.

### Increased Constitutive PEK-1 Activation in *C. elegans xbp-1* Mutants

**Figure 2:**
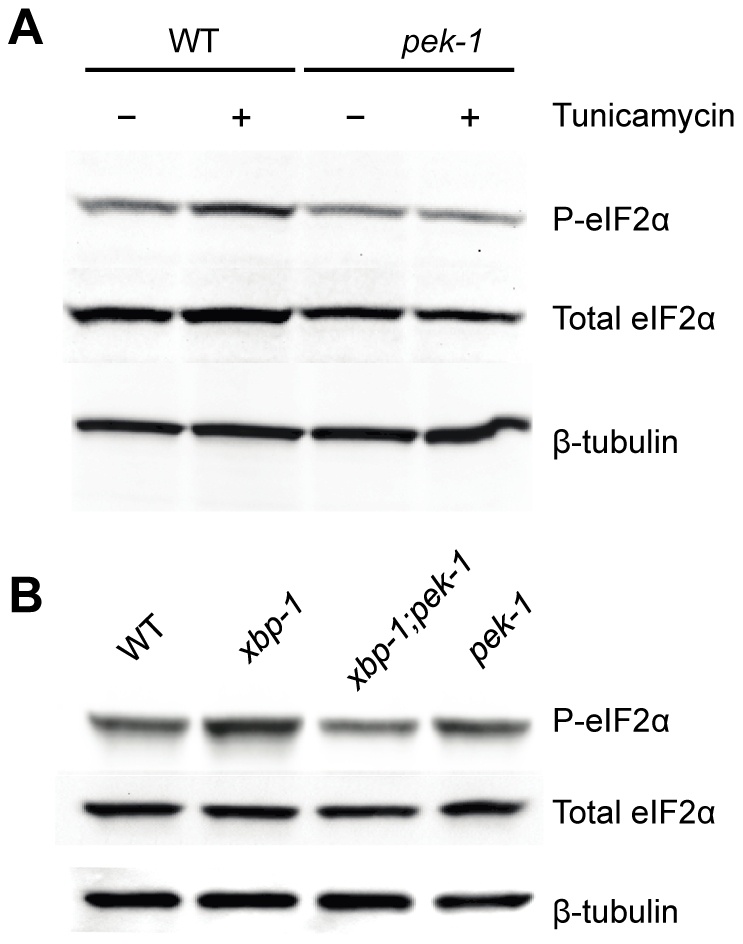
XBP-1 deficiency increases PEK-1 dependent phosphorylation of eIF2α. (A) PEK-1 dependent phosphorylation of eIF2α is induced by high dose (50 ug/ml) tunicamycin. Western blot of P-eIF2α, total eIF2α and tubulin in WT and *pek-1(ok275*) strains grown to the L4 stage and then shifted to plates with or without tunicamycin (50 µg/mL) for 4 hours. (B) PEK-1 dependent phosphorylation of eIF2α is induced by XBP-1 deficiency. Western blot of P-eIF2α, total eIF2α and tubulin in WT, *xbp-1(tm2482)*, *xbp-1(tm2482)*; *pek-1(ok275)* and *pek-1(ok275*) strains grown to the L4 stage.

If the elevated level of IRE-1 activity observed in the *smg-2(qd101)*; *xbp-1(zc12)* mutant were indicative of increased ER stress due to loss of *xbp-1*, we might anticipate compensatory activation of the PEK-1 and/or ATF-6 pathways in the absence of XBP-1. We therefore sought to determine levels of PEK-1 activity in an *xbp-1* mutant through the detection of eIF-2α phosphorylation. In particular, these measurements would provide an additional measure of ER stress in the xbp-1 mutant that is not dependent on the inactivation of the NMD pathway and its aforementioned experimental caveats. Antibodies raised against mammalian eIF-2α and specifically phosphorylated eIF-2α (P-eIF-2α) cross-react with the highly homologous *C. elegans* protein [25], [26]. We detected a single band in immunoblots using these antibodies with lysates from WT *C. elegans* (Figure 2A). We observed that eIF-2α phosphorylation was induced by a 4 h exposure to a high dose of tunicamycin in a PEK-1-dependent manner (Figure 2A and Figure S1A). Of note, eIF-2α phosphorylation appears to be a less sensitive measure of ER stress than IRE-1-mediated *xbp-1* mRNA splicing, as we did not observe a significant increase in eIF-2α phosphorylation in response to standard doses of tunicamycin sufficient to induce *xbp-1* mRNA splicing.

We next determined PEK-1 activity under basal physiological conditions, specifically in the *xbp-1* mutant. We saw induction of PEK-1-mediated eIF-2α phosphorylation relative to WT in the absence of exogenously administered agents to induce ER stress at 16°C (Figure 2B and Figure S1B). The magnitude of the effect of XBP-1 deficiency on PEK-1 activity was comparable to the induction of PEK-1 in WT by treatment with high-dose tunicamycin. We observe no increase in eIF-2α phosphorylation in the *xbp-1*; *pek-1* mutant relative to the *pek-1* mutant, confirming that the increase in eIF-2α phosphorylation in the *xbp-1* mutant relative to WT is due to activation of PEK-1. In fact, we noticed a slight decrease in eIF-2α phosphorylation in the *xbp-1*; *pek-1* mutant relative to the *pek-1* mutant, but the mechanisms underlying this difference are unclear. These data corroborate our observations of increased *xbp-1* mRNA splicing in the *xbp-1* mutant. Taken together, the increase in levels of IRE-1 and PEK-1 activity in the *xbp-1* mutant suggests that XBP-1 deficiency is accompanied by a marked increase in constitutive ER stress under basal physiological conditions.

### The Effects of Immune Activation on ER Stress Levels in the *xbp-1* Mutant

Previously, we reported that the activation of innate immunity by infection with pathogenic *P. aeruginosa* induces ER stress, and that XBP-1 serves an essential role in protecting the host against the detrimental effects of immune activation [21]. Our prior ultrastructural analysis of the ER in *xbp-1* mutants suggested that disruption of ER homeostasis contributes to this phenotype. One explanation for these observations is that ER homeostasis in the *xbp-1* mutant might be minimally perturbed under basal physiological conditions but have a pronounced sensitivity to ER stress from endogenous (e.g. immune activation) or exogenous (e.g. tunicamycin) sources. However, the data in Figure 1 and Figure 2 suggest that even during physiological growth and development, XBP-1 deficiency results in a marked elevation in levels of basal ER stress. We hypothesized, therefore, that under these circumstances, the activation of innate immunity might further increase ER stress levels.

**Figure 3:**
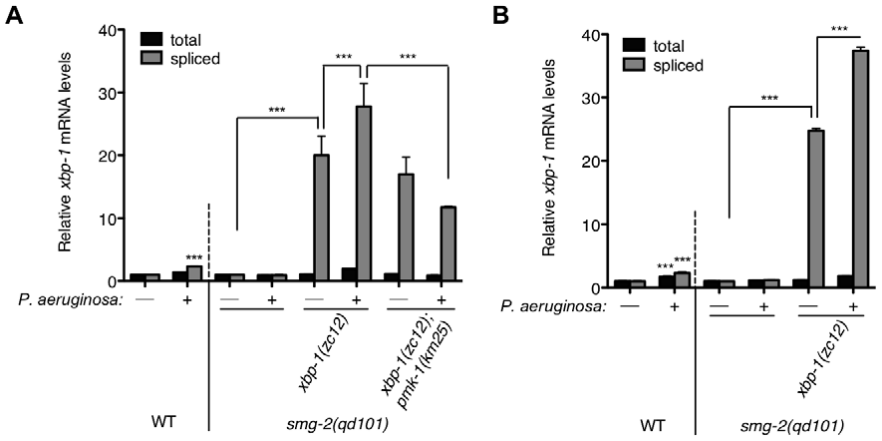
Pathogen-induced immune activation exacerbates ER stress levels in XBP-1 deficiency in *C. elegans*. Quantitative real-time PCR measurements of levels of total and spliced *xbp-1* mRNA in the indicated strains grown from synchronized L1s for 23 h at 20°C and then shifted to plates with or without *P. aeruginos*a PA14 at 25°C for (A) 4 h or (B) 11 h. Values represent fold change ± s.e.m. (n = 2 independent experiments, ***P<0.001, two-way ANOVA with Bonferroni post test). The WT strain exposed to either treatment was normalized to WT without *P. aeruginosa*; all other strains were normalized to *smg-2(qd101)* without *P. aeruginosa*.

The *smg-2(qd101)*; *xbp-1(zc12)* strain provided the opportunity to assess levels of ER stress caused by immune activation in the setting of XBP-1 deficiency. Whereas a 4 h exposure of the WT strain to *P. aeruginosa* PA14 causes a two-fold increase in spliced *xbp-1* mRNA relative to exposure to the relatively non-pathogenic bacterial food *Escherichia coli* OP50 (Figure 3A and [21]), we observe a blunted response to *P. aeruginosa* infection in the *smg-2(qd101)* mutant (Figure 3A). This observation is likely due to the 5-fold elevation in spliced *xbp-1* mRNA levels in the *smg-2(qd101)* mutant (Figure 1C), which may buffer the ER from the stress caused by pathogen-induced immune activation. Nevertheless, we observed that the level of spliced *xbp-1* mRNA in the *smg-2(qd101)*; *xbp-1(zc12)* mutant was increased by a 4 h exposure to *P. aeruginosa* relative to the *smg-2(qd101)*; *xbp-1(zc12)* mutant treated in parallel with *E. coli* (Figure 3A). Specifically, under these treatment conditions, the level of spliced *xbp*-1 mRNA in the smg-*2(qd101)*; *xbp-1(zc12)* mutant was 20-fold greater than that of the *smg-2(qd101)* mutant in the absence of additional stress, whereas exposure to *P. aeruginosa* increased the level of spliced *xbp-1* mRNA to over 25-fold that of the *smg-2(qd101)* mutant (Figure 3A). The total amount of *xbp-1* mRNA was unchanged between *smg-2(qd101)* and *smg-2(qd101)*; *xbp-1(zc12)* strains, indicating that the increase in spliced *xbp-1* mRNA is due to increased IRE-1 activation.

We observed persistent elevation of spliced *xbp-1* mRNA after an 11 h exposure to *P. aeruginosa*, above the elevated basal levels of spliced *xbp-1* mRNA in the *xbp-1* mutant, suggesting that IRE-1 activity is not attenuated under conditions of physiological ER stress (Figure 3B).

Previously, we established that the ER stress induced by exposure to *P. aeruginosa*, as well as the lethality of the *xbp-1* mutant during infection by *P. aeruginosa*, are suppressed by a loss-of-function mutation in *pmk-1*, which encodes a conserved p38 mitogen-activated protein kinase (MAPK) that regulates innate immunity in *C. elegans* [27]. Our interpretation of these data was that loss of PMK-1 activity diminished the secretory load on the ER by attenuating the innate immune response. In support of this interpretation, we found that the pathogen-induced increase in spliced *xbp-1* mRNA in *smg-2(qd101)*; *xbp-1(zc12)* was suppressed in the *smg-2(qd101)*; *xbp-1(zc12)*; *pmk-1(km25)* mutant, although the basal levels on *E. coli* OP50 nevertheless remained markedly elevated (Figure 3A). These data provide quantitative support for a model in which the activation of PMK-1-mediated innate immunity is a physiologically relevant source of ER stress, which in XBP-1-deficient animals exacerbates an already elevated level of ER stress to cause larval lethality.

### A Temperature-Dependent Requirement for XBP-1 and PEK-1 Activities in *C. elegans* Development and Survival

What are the functional consequences of the elevated ER stress present in the *xbp-1* mutant under standard growth conditions, in the absence of infection? The *xbp-1* mutant, while viable, exhibits increased sensitivity to exogenously administered ER stress as well as physiological ER stress from immune activation [21], [28]. Inactivation of both *xbp-1* and *pek-1* was previously reported to result in larval arrest when propagated at 20°C [16]. Our observations of constitutive ER stress in the *xbp-1* mutant and increased PEK-1 activity suggest a compensatory functional role for *pek-1*, and thus we sought to further characterize the larval arrest phenotype of the *xbp-1*; *pek-1* mutant.

**Figure 4:**
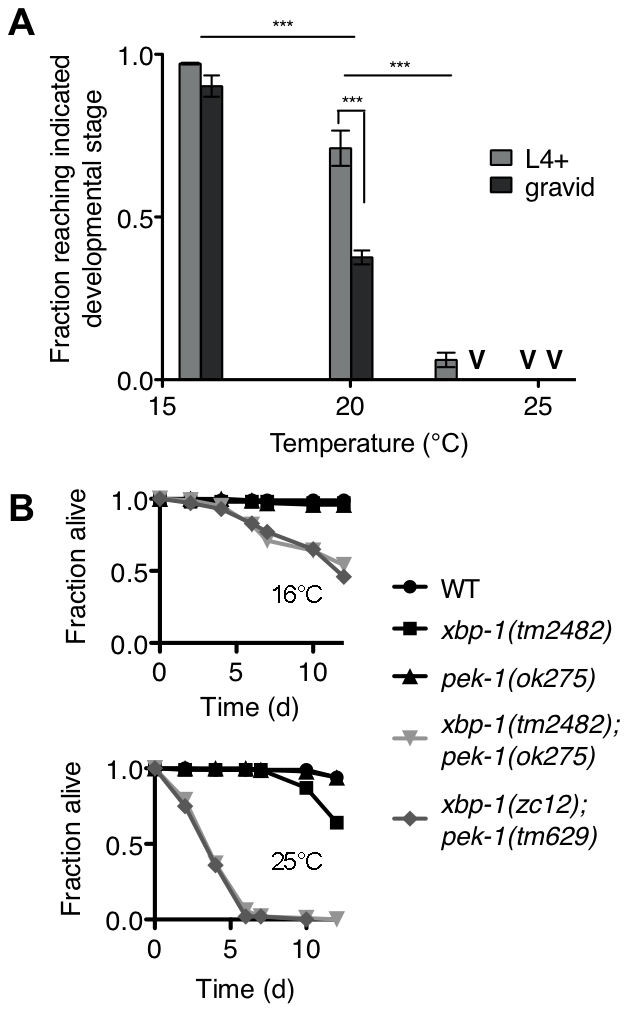
Temperature-sensitive lethality of the *xbp-1;pek-1* double mutant. (A) Development of the *xbp-1(tm2482)*; *pek-1(ok275)* mutant across physiological temperatures. Values represent average fraction of eggs developed to the indicated stage ± s.e.m. (n = 3 independent experiments at 20°C, 2 independent experiments at all other temperatures; ***P<0.001, two-way ANOVA with Bonferroni post test). (B) Lifespan of indicated strains grown at 16°C to the L4 stage, then shifted to either 16°C to 25°C. Results are representative of two independent experiments.

Surprisingly, we observed that the *xbp-1(tm2482)*; *pek-1(ok275)* double mutant exhibited temperature-dependent viability over the physiological temperature range of *C. elegans* (Figure 4A). The larval development of the *xbp-1(tm2482)*; *pek-1(ok275)* mutant was similar to that of WT at 16°C. At 20°C, however, approximately half of *xbp-1(tm2482)*; *pek-1(ok275)* eggs developed to become gravid adults, while the remainder arrested during larval development in the L2 and L3 stages. These arrested larvae died over the course of several days with intestinal degeneration as previously described (Shen et al., 2005). At temperatures greater than 23°C, larval lethality was 100%. At 25°C, 100% of the population died in the L1 and L2 stages after just 2 days. The physiological temperature range for propagation of *C. elegans* in the laboratory is generally 15°C to 25°C, with optimal reproduction at 20°C. Thus, the observed temperature dependence is observed not at “heat shock” temperatures, but rather, well within the range of physiological temperatures for *C. elegans*.

The temperature dependence of *xbp-1(tm2482)*; *pek-1(ok275)* lethality permitted the investigation of whether the larval lethality of the *xbp-1*; *pek-1* mutant is due to a requirement for XBP-1 and PEK-1 at a specific stage of development, or whether the activities of XBP-1 and PEK-1 are required constitutively for viability at other life stages. Specifically, we propagated two *xbp-1*; *pek-1* mutants comprised of different mutant alleles at 16°C until the animals reached the L4 larval stage, then either maintained the mutants at 16°C or shifted them to 25°C to monitor survival. When shifted to 25°C, the *xbp-1*; *pek-1* double mutants exhibited a sharp decrease in survival as compared with the strains maintained at 16°C (Figure 4B). These observations suggest that the activity of either XBP-1 or PEK-1 is not specifically required at a particular developmental stage; instead, the constitutive activities of XBP-1 and PEK-1 are required for survival at physiological temperatures.

### XBP-1 and PEK-1 Maintain Intestinal Cell Homeostasis during ER Stress Caused by Basal and Induced Innate Immunity

**Figure 5:**
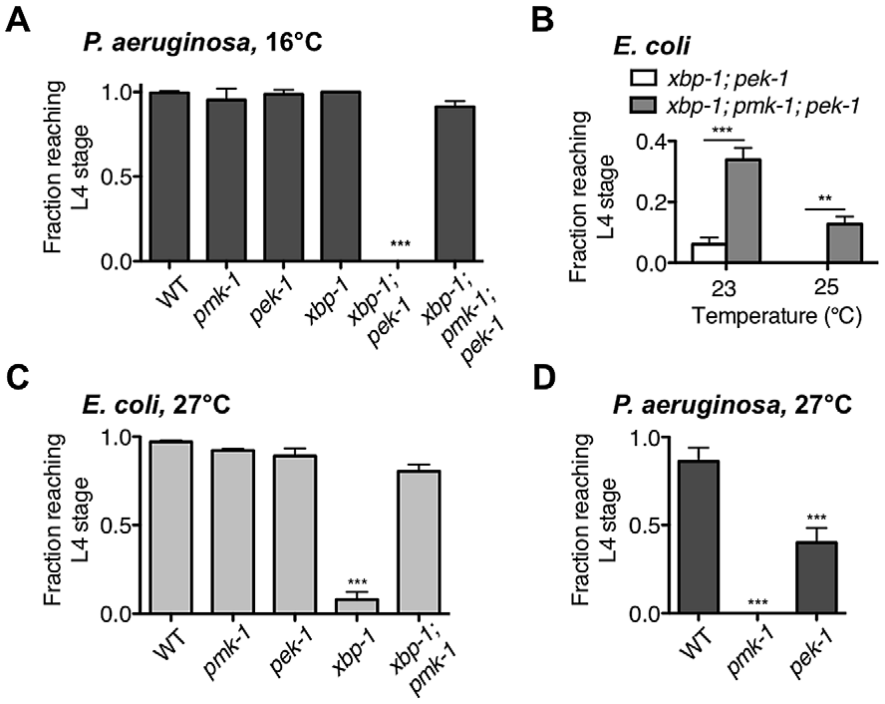
XBP-1 and PEK-1 each protect against elevated physiological temperature and immune activity. (A) Development of indicated mutants from eggs to the L4 larval stage or older after 4 d at 16°C on *P. aeruginosa* PA14. Values represent mean ± s.d. from 1 of 2 representative experiments (n = 4 plates with 20–50 eggs each, ***P<0.001, one-way ANOVA with Bonferroni post test). (B) Development of *xbp-1(tm2482)*; *pek-1(ok275)* and *xbp-1(tm2482)*; *pmk-1(km25)*; *pek-1(ok275)* mutants from eggs on *E. coli* OP50. Values represent average fraction of eggs developed to the indicated stage ± s.e.m. (n = 2 independent experiments, **P<0.01, ***P<0.001, two-way ANOVA with Bonferroni post test). (C) Development of indicated mutants from eggs to the L4 larval stage or older after 2 d at 27°C on *E. coli* OP50. Values represent mean ± s.e.m. (n = 3 independent experiments, ***P<0.001, one-way ANOVA with Bonferroni post test). (D) Development of indicated mutants from eggs to the L4 larval stage or older after 2 d at 27°C on *P. aeruginosa* PA14. Values represent mean ± s.d. from 1 of 2 representative experiments (n = 3–4 plates with 20–60 eggs each, ***P<0.001, one-way ANOVA with Bonferroni post test).

Although we previously observed that *pek-1* and *atf-6* single mutants did not exhibit larval lethality in the presence of pathogenic bacteria [21], our data presented in this paper suggest that PEK-1 functions in parallel to XBP-1 under physiological conditions in *C. elegans* to maintain ER homeostasis. Because the *xbp-1*; *pek-1* mutant is viable through larval development at 16°C, we were able to ask whether PEK-1 contributes to protection against immune activation in the absence of XBP-1. Populations of synchronized eggs were grown at 16°C with *P. aeruginosa* as the only food source and development was monitored over time. *P. aeruginosa* has been shown to exhibit markedly diminished pathogenicity to *C. elegans* adults at 16°C relative to 25°C [29], and we found this to also be the case during larval development. Specifically, the *pmk-1* mutant was able to complete larval development on *P. aeruginosa* at 16°C (Figure 5A), whereas only half of the *pmk-1* eggs grown on *P. aeruginosa* develop to the L4 stage at 25°C [21], indicating that immune activation is less important for development in the presence of *P. aeruginosa* grown at 16°C than it is at 25°C. Likewise, the larval development of the *xbp-1* mutant, which is severely compromised on *P. aeruginosa* at 25°C [21], was equivalent to that of WT at 16°C (Figure 5A). Both the diminished pathogenicity of *P. aeruginosa* at 16°C and the aforementioned temperature-sensitive requirement for UPR function may contribute the survival of the *xbp-1* mutant at 16°C. Nevertheless, even under these conditions, the *xbp-1(tm2482)*; *pek-1(ok275)* mutant exhibited complete larval lethality on *P. aeruginosa* at 16°C, reminiscent of the larval lethality of *xbp-1* on *P. aeruginosa* grown at 25°C. Eliminating PMK-1-mediated immunity completely rescued this larval lethality (Figure 5A), demonstrating that PEK-1 functions with XBP-1 to protect against PMK-1-mediated immune activation during larval development.

We next asked whether the UPR is required for survival in the presence of pathogen during adulthood. In parallel with our observation that the *xbp-1*; *pek-1* mutant exhibits temperature-sensitive lethality both during larval development and when shifted to a higher temperature from the L4 larval stage, we found that the *xbp-1*; *pek-1* mutant exhibits enhanced lethality relative to the WT strain or either of the single mutants when shifted at the L4 stage to *P. aeruginosa* at 16°C (Figure S2). These data suggest that the UPR is required for survival during immune activation both in larval development and in adulthood.

Larval arrest of *xbp-1*; *pek-1* mutants has been reported to be accompanied by evidence of intestinal degeneration, including the appearance of vacuoles and light-reflective aggregates in intestinal cells, degradation of intestinal tissues, and distention of the intestinal lumen [16]. We observe similar morphology not only in *xbp-1*; *pek-1* larvae at 23°C on *E. coli* OP50, but also at 16°C on *P. aeruginosa*. The similar appearance between *xbp-1*; *pek-1* larvae dying either at 16°C on pathogenic bacteria or at 23°C on *E. coli* OP50 led us to consider whether ER stress arising from intestinal innate immune activation might contribute in a similar manner to both conditions. We have previously characterized PMK-1-mediated innate immunity and observed both basal and induced components to immunity regulated by PMK-1 [30]. We therefore hypothesized that basal immune activity under standard, non-pathogenic growth conditions could present a low level of ER stress that is severely exacerbated in the absence of intact physiological UPR function, leading to larval lethality of the *xbp-1*; *pek-1* mutants. Consistent with this hypothesis, we observed that *pmk-1* loss-of-function was able to partially suppress the larval lethality of the *xbp-1*; *pek-1* double mutant at 23°C and 25°C (Figure 5B).

One explanation for the temperature-sensitive lethality of the *xbp-1*; *pek-1* mutant is that increased temperature leads to increased PMK-1 pathway activation, perhaps as the “non-pathogenic” *E. coli* OP50 becomes slightly pathogenic. However, the temperature-sensitive lethality is not abrogated by loss of PMK-1; instead, the *xbp-1*; *pmk-1*; *pek-1* mutant exhibits larval lethality at a temperature several degrees higher than the *xbp-1*; *pek-1* mutant (Figure 5B). Furthermore, the temperature-sensitive larval lethality of the *xbp-1*; *pek-1* mutant on *E. coli* OP50 was not suppressed by the presence of the bacteriostatic drug ampicillin (Figure S3A). These data indicate that basal immune activation and temperature are distinct contributors to ER stress that function in parallel during growth on *E. coli* OP50.

### Thermal Stress Necessitates UPR Signaling for Survival in *C. elegans*

The temperature-dependent larval lethality of the *xbp-1*; *pek-1* mutant over a physiological temperature range suggested that UPR signaling might be required for survival in response to thermal stress. Indeed, we observed that the *xbp-1* mutant exhibited larval lethality when grown at 27°C, an elevated temperature at which WT N2 *C. elegans* exhibits a reduced brood size and increased dauer formation (Figure 5C). Similar to our observation that depletion of basal immunity rescued the development of the *xbp-1*; *pek-1* mutant when propagated on *E. coli* OP50, the temperature-sensitive lethality in the *xbp-1* mutant was suppressed in the *xbp-1*; *pmk-1* double mutant (Figure 5C), but not by the presence of ampicillin (Figure S3B).

Unlike the *xbp-1* mutant, the development of the *pek-1* mutant at 27°C was similar to WT. This is reminiscent of our previous observation that the *pek-1* mutant did not exhibit the larval lethality found in *xbp-1* when grown on *P. aeruginosa* at 25°C [21]. However, we next grew the *pek-1* mutant on *P. aeruginosa* at 27°C, reasoning that the elevated temperature would not only increase the ER stress caused by basal growth, but also enhance the pathogenicity of the *P. aeruginosa* and thereby increase the immune response relative to that at 25°C. Indeed, the *pmk-1* mutant exhibited 100% larval lethality on *P. aeruginosa* at 27°C (Figure 5D), as compared with the 50% lethality we have previously reported for the *pmk-1* mutant on *P. aeruginosa* at 25°C (Richardson et al., 2010). The increased susceptibility of this immune-deficient mutant to *P. aeruginosa* at 27°C relative to 25°C indicates that the increased temperature causes an increase in *P. aeruginosa* pathogenicity. On *P. aeruginosa* at 27°C, the *pek-1* mutant exhibited larval lethality relative to the WT strain grown 27°C (Figure 5D). These data further suggest that PEK-1 functions in parallel with XBP-1 to protect *C. elegans* against the ER stress caused by immune activation.

### The PMK-1 Pathway Protects Against Exogenous ER Stress

**Figure 6:**
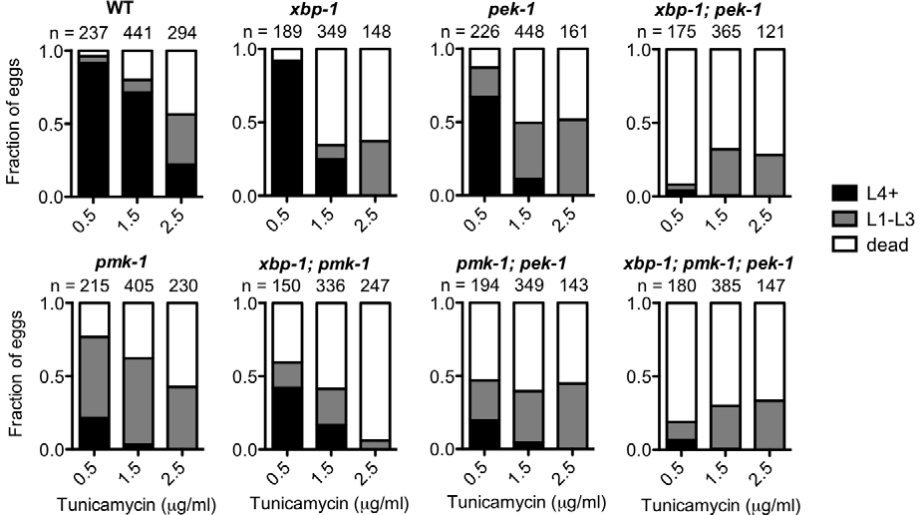
PMK-1 protects against exogenously induced ER stress. Larval development and survival assay showing the proportion of animals of each of the indicated strains that reach the indicated stage after 4 d of development from eggs laid on plates containing tunicamycin at 16°C. Values are from either one experiment (0.5 µg/ml and 2.5 µg/ml tunicamycin) or combined from two experiments with similar results (1.5 µg/ml tunicamycin).

We showed in Figure 5B and 5C that loss of PMK-1 improves larval development of the *xbp-1*; *pek-1* mutant and the *xbp-1* mutant, respectively, in the absence of infection. We suggested that the mechanism behind this phenomenon is that the previously described basal immune activity through the PMK-1 pathway [31] contributes to ER stress. However, we also considered the possibility that the PMK-1 pathway might play an immunity-independent role in exacerbating ER stress in the setting of UPR deficiency. To test this possibility, we examined the ability of WT and UPR mutants to develop in the presence of tunicamycin with or without functional *pmk-1*. We found that the *pmk-1* mutant actually exhibited increased sensitivity to tunicamycin during development. In fact, the *pmk-1* mutant exhibited greater lethality at a lower dose of tunicamycin than either the *xbp-1* or *pek-1* single mutants (Figure 6). These data suggest that the PMK-1 pathway influences ER stress in two ways. First, during infection or under standard growth conditions in the setting of UPR depletion, activation of the PMK-1 pathway generates an increased secretory load that contributes to ER stress. However, when ER stress is induced exogenously with tunicamycin, the PMK-1 pathway activity serves a protective function.

## Discussion

We have shown that the IRE-1-XBP-1 and PEK-1 pathways function together to maintain ER homeostasis in *C. elegans* under physiological conditions. We found that XBP-1 deficiency results in marked activation of both IRE-1 and PEK-1, reflecting constitutive ER stress. Activation of innate immunity mediated by PMK-1 p38 MAPK further exacerbated the constitutive ER stress in the *xbp*-1 mutant. To investigate the physiological roles of UPR signaling as well as the compensatory activity between distinct UPR pathways, we examined both the individual and the combined effects of XBP-1 and PEK-1 deficiency *in vivo*. We found that the *xbp-1*; *pek-1* double mutant exhibited temperature-sensitive lethality that was independent of developmental stage. Compared with the *xbp-1*; *pek-1* mutant, the *xbp-1*; *pmk-1*; *pek-1* mutant had moderately increased survival during larval development on non-pathogenic bacteria, when there is a low level of PMK-1-mediated basal immune activity, and dramatically increased survival on pathogenic *P. aeruginosa*, when the PMK-1-mediated immune response is induced. We further showed that both XBP-1 and PEK-1 are required for full protection against the combined stress of immune activation and that of growth at elevated physiological temperatures, confirming that these two branches of the UPR function together to protect against physiological ER stress.

**Figure 7:**
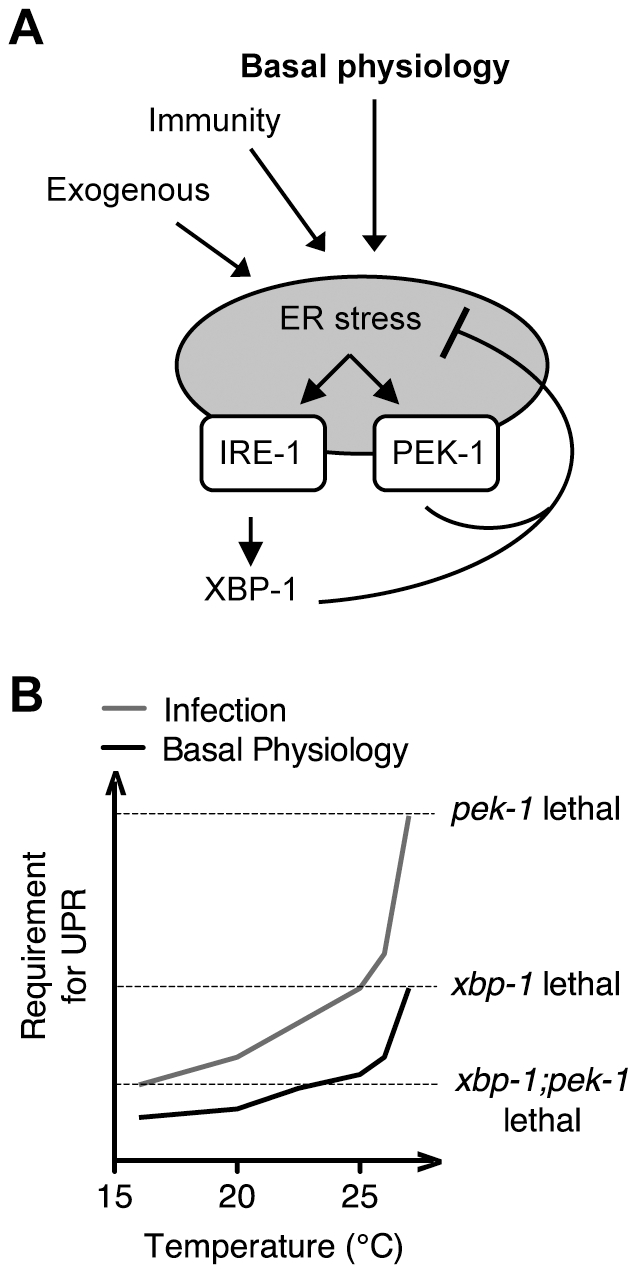
Maintenance of ER homeostasis through activation of the IRE-1 and PEK-1 pathways under basal physiological conditions during development. (A) The increase in both IRE-1 and PEK-1 activities in XBP-1 deficiency in the absence of exogenous compounds to impose ER stress, combined with the temperature-sensitive lethality of the UPR mutants, implies that UPR signaling maintains ER homeostasis not only in response to the extremes of ER stress, but also under basal physiological conditions. (B) Infection, basal growth and development, and elevated physiological temperature all contribute to ER stress, leading to lethality of UPR mutants as indicated by dashed lines.

Our observation of dramatically elevated levels of IRE-1 and PEK-1 activity in the setting of XBP-1 deficiency, under standard growth conditions in the absence of exogenous agents to induce ER stress, provides strong evidence for homeostatic activity of the IRE-1-XBP-1 signaling pathway under physiological conditions (Figure 7A), and not merely at the extremes of ER stress induced by pharmacological treatment or in specialized secretory cell types. Our data also reveal a dynamic requirement for UPR signaling in survival that increases with both temperature and increased secretory activity as is induced by immune activation (Figure 7B). Interestingly, the temperature-dependent role for the IRE-1 and PEK-1 pathways is manifest at physiological temperatures optimal for *C. elegans* development and fecundity, far from commonly utilized “heat shock” conditions (Figure 7A). We speculate that this temperature dependence may be due to altered secretory load at higher temperature or increased tendency for proteins to aggregate in the ER in the absence of intact chaperone production.

Importantly, our data suggest that PMK-1-mediated immune activation is one of many sources of the requirement for the UPR during larval development in the absence of infection. We found that, although loss of basal PMK-1 pathway activation partially suppressed the temperature-sensitive larval lethality of the *xbp-1*; *pek-1* mutant, the *xbp-1*; *pmk-1*; *pek-1* mutant nevertheless exhibited almost complete larval lethality at 25°C. Further, using our *smg-2(qd101)*; *xbp-1(zc12)* strains, we observed high constitutive IRE-1-mediated *xbp-1* splicing in the *xbp-1*; *pmk-1* mutant that was similar under these experimental conditions to that of the *xbp-1* mutant (Figure 3A). These results indicate that the UPR has an essential role during development in protection against immune activation as well as additional processes. Identification of these processes will likely lead to increased understanding of conserved physiological roles of the UPR.

We found that the PMK-1 pathway not only contributes to basal ER stress but also protects against exogenous ER stress induced by exposure to tunicamycin (Figure 6). We speculate that the mechanism underlying this dual function of the PMK-1 pathway may be differences in the PMK-1-activated transcriptional output under different circumstances. The importance of the PMK-1 pathway in protection against exogenous ER stress makes the role of the PMK-1 pathway in contributing to endogenous ER stress even more striking.

In mice, *Xbp1* deficiency in intestinal epithelial cells (IEC) resulted in marked intestinal inflammation that may contribute to the observed activation of not only IRE1 but also of PERK, as measured by expression of one of its downstream effectors, CHOP [12]. In mammals, the transcription factor CHOP promotes apoptosis of mammalian cells that experience prolonged ER stress [32], and indeed, the majority of Paneth cells underwent apoptosis in the *Xbp1^−/−^* IECs. Our observations are consistent with the idea that *Xbp1^−/−^* IECs may be predisposed to detrimental consequences of additional ER stress caused by intestinal inflammation because of deregulation of basal ER homeostasis due to XBP-1 deficiency. In pancreatic ß-cells, another cell type that is specialized for high-level secretory activity, *XBP1* deficiency has been observed to result in *IRE1*α hyperactivation, with increased degradation of mRNAs that encode insulin processing enzymes [33].

Our observations that PEK-1, in concert with XBP-1, functions to protect against ER stress from immune activation differ from observations in mouse macrophages, in which TLR stimulation was shown to activate IRE1, but PERK activation was reported to be suppressed rather than elevated [34], [20]. This difference may be due to roles for XBP-1 in macrophages that extend beyond its function in maintaining ER homeostasis. Indeed, when stimulated by TLRs in macrophages, the IRE1-XBP1 pathway was shown to induce expression of immune effectors rather than typical UPR genes, suggestive that the IRE1-XBP1 pathway may have been co-opted in macrophages to promote macrophage-specific function independent of the UPR [20].

Our data support the idea that UPR signaling does not function simply in response to the extremes of ER stress, as when induced by tunicamycin or by the elevated secretory load of specialized cells such as plasma cells, but instead, as a critical pathway in the maintenance of ER homeostasis during normal growth and development in *C. elegans*. The diverse and dramatic consequences of XBP-1 deficiency on development and disease, taken together with our observations on the effect of XBP-1 deficiency on basal ER stress levels, underscore the critical role of homeostatic UPR signaling in both normal physiology and disease.

## Materials and Methods

### Strains

*C. elegans* strains were constructed and propagated according to standard methods on *E. coli* OP50 at 16°C [35]. The *smg-2(qd101)* allele was isolated by K. Reddy and contains a C→T nonsense mutation at nucleotide 1189 of the spliced transcript. The following strains were used in the study: N2 Bristol, ZD627 *smg-2(qd101)*, ZD607 *smg-2(qd101);xbp-1(zc12)*, ZD605 *smg-2(qd101);xbp-1(zc12);pmk-1(km25)*, KU25 *pmk-1(km25)*, RB545 *pek-1(ok275)*, ZD510 *xbp-1(tm2482);pek-1(ok275)*, ZD524 *xbp-1(zc12);pek-1(tm629)*, ZD496 *xbp-1(tm2482);pmk-1(km25);pek-1(ok275)*. All of the alleles used are predicted to be null alleles. Specifically, *xbp-1(tm2482)* is a 202 bp deletion from nt 231 that causes a frame-shift. The *xbp-1(zc12)* allele is a nonsense mutation that changes Q34 to an ochre stop. The two alleles exhibit an equivalent phenotype in every assay tested ([21]; this work, and our unpublished data). The *pek-1(ok275)* allele is a 2013 bp deletion and the *pek-1(tm629)* allele is a 1473 bp deletion, both of which remove the PEK-1 transmembrane domain and are therefore likely null alleles [6]. Double mutants were made between *xbp-1* and *pek-1* by crossing strains marked with GFP: *xbp-1*(III)*;pT24B8.5::GFP(agIs220)*(X) and *pT24B8.5::GFP(agIs219)*(III);*pek-1*(X). GFP-negative F2s were singled, propagated, and genotyped by PCR.

### RNA Isolation and Quantitative RT-PCR

For the experiment in Figure 1B, 1C, and 1E, L1 larvae were synchronized by hypochlorite treatment, washed onto *E. coli* OP50 plates, and grown for 40 h at 16°C to the L3 stage, when they were washed in M9 to plates containing *E. coli* OP50 or *E. coli* OP50 with 5 µg/ml tunicamycin. For the experiments in Figure 3, strains were grown and treated as previously described [21]. Specifically, L1 larvae were synchronized by hypochlorite treatment, washed onto *E. coli* OP50 plates and grown at 20°C for 23 h, then washed in M9 onto treatment plates. After incubation at 25°C for indicated times, worms were washed off plates and frozen in liquid nitrogen. For *P. aeruginosa* treatment, *P. aeruginosa* strain PA14 was grown in Luria Broth (LB), and 25 µl overnight culture was seeded onto 10 cm NGM plates. Plates were incubated first at 37°C for 1 d, then at room temperature for 1 d. All RNA extraction, cDNA preparation, qRT-PCR methods and specific primers to detect *xbp-1* mRNA were as described previously [21].

### Immunoblotting

For all immunoblots, strains were synchronized by hypochlorite treatment and washed onto *E.coli* OP50 plates for growth until the L4 stage. For the experiment in Figure 2A, strains were grown at 20°C and L4 worms were then washed in M9 onto treatment plates for incubation at 25°C for 4 hours. For the experiment in Figure 2B, strains were grown at 16°C until the L4 stage and harvested without treatment. All strains were collected and rinsed 2 times in M9. Worm pellets were resuspended in an equal volume of 2× lysis buffer containing 4% SDS, 1oomM Tris Cl, pH 6.8, and 20% Glycerol. After boiling for 15 minutes with occasional vortexing to aid in dissolution, lysates were clarified by centrifugation. Protein samples (50 µg of total lysate loaded per lane) were separated by SDS-PAGE and transferred to a nitrocellulose membrane (Bio-rad). Western blots were blocked in 5% milk in PBST and probed with (1∶10,000) anti-eIF2α [26], (1∶1,000) anti-phospho-eIFα (Cell Signaling Technology), or (1∶10,000) anti-tubulin (E7 Developmental Hybridoma Bank, Iowa City). All primary antibodies were diluted in 5% milk in PBST. Following incubation with anti-rabbit or anti-mouse IgG antibodies conjugated with horseradish peroxidase (HRP) (Cell Signaling Technology), signals were visualized with chemiluminescent HRP substrate (Amersham). Quantification of immunoblots was preformed with ImageJ [36].

### Survival/Development Assays

For all development assays, strains were egg laid on 4–5 prepared plates for no more than 3 h (at least 110 eggs for each strain and treatment). Development was monitored daily for 4 d for experiments conducted at 16°C and 3 d for experiments conducted at all other temperatures. Experiments monitoring development on *E. coli* OP50 were performed on 6 cm NGM plates. *P. aeruginosa* PA14 plates were prepared as described [27]. For the data presented in Figure S2, plates were prepared as described [37], except that ampicillin was used instead of carbenicillin.

To monitor L4 survival on *E. coli* OP50 or *P. aeruginosa* PA14, strains were incubated at 16°C to the L4 stage, when they were transferred to plates containing FUDR and incubated at either 16°C or 25°C. For each strain, 30 worms were transferred to each of 3–4 plates. Alive vs. dead worms were counted, and worms that died by exploding through the vulva or desiccating on the side of plates were censored.

## Supporting Information

Figure S1 Exposure to tunicamycin or XBP-1 deficiency increases PEK-1 dependent phosphorylation of eIF2α. Quantification of immunoblots presented in (A) Figure 2A and (B) Figure 2B. Band intensity for P-eIF2α and total eIF2α was normalized to that of ß–tubulin for each strain, and values represent fold change relative to WT.
(TIFF)

Figure S2 The UPR protects against lethality in the presence of pathogen during adulthood. Survival of WT, *xbp-1(tm2482)*, *pek-1(ok275)*, and *xbp-1(tm2482)*; *pek-1(ok275)* strains grown at 16°C to the L4 stage, then shifted to plates seeded with *P. aeruginosa* PA14. Results are representative of two independent experiments.
(TIFF)

Figure S3 Temperature-sensitive lethality of the *xbp-1*; *pek-1* mutant is not caused by increased pathogenicity of *E. coli* OP50 at elevated temperatures. (A) Development of *xbp-1(tm2482)*; *pek-1(ok275)* mutant from eggs on *E. coli* OP50 after 3 d at 23°C with or without the bacteriostatic drug ampicillin. (B) Development of the N2 WT strain and the *xbp-1(tm2482)* mutant from eggs on *E. coli* OP50 after 3 d at 27°C with or without the bacteriostatic drug ampicillin. Values represent average fraction of eggs developed to the L4 larval stage or later ± s.e.m. (n = 2 independent experiments).
(TIFF)
