# Supplementary figures and images for: Physiological IRE-1-XBP-1 and PEK-1 Signaling in Caenorhabditis elegans Larval Development and Immunity
Source: PLoS Genet. 2011 Nov 17;7(11):e1002391. doi: 10.1371/journal.pgen.1002391 (PMC3219621; doi:10.1371/journal.pgen.1002391)

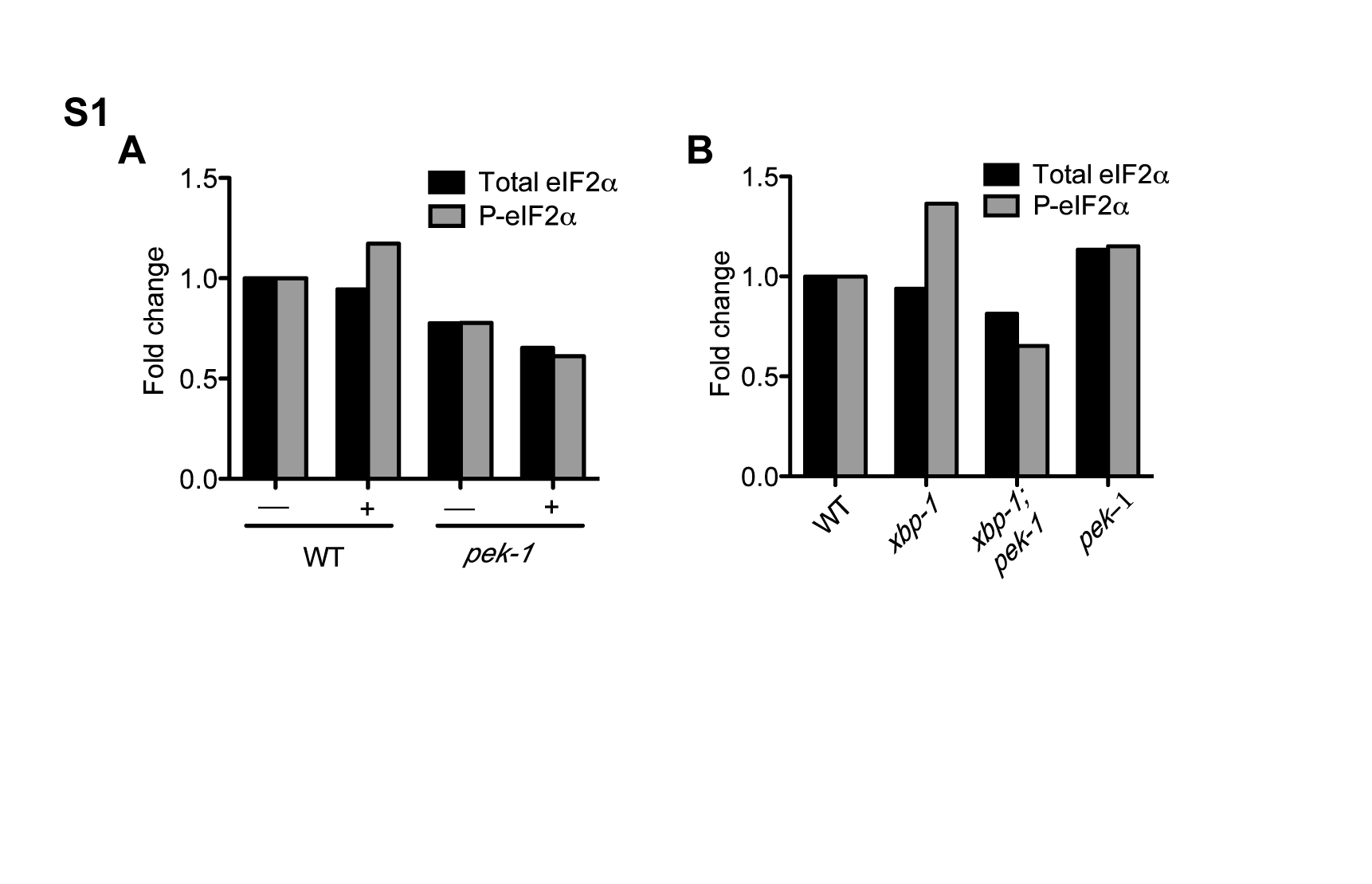

Supplement: Figure S1 — Exposure to tunicamycin or XBP-1 deficiency increases PEK-1 dependent phosphorylation of eIF2α. Quantification of immunoblots presented in (A) Figure 2A and (B) Figure 2B. Band intensity for P-eIF2α and total eIF2α was normalized to that of ß–tubulin for each strain, and values represent fold change relative to WT. (TIFF) [file pgen.1002391.s001.tiff]

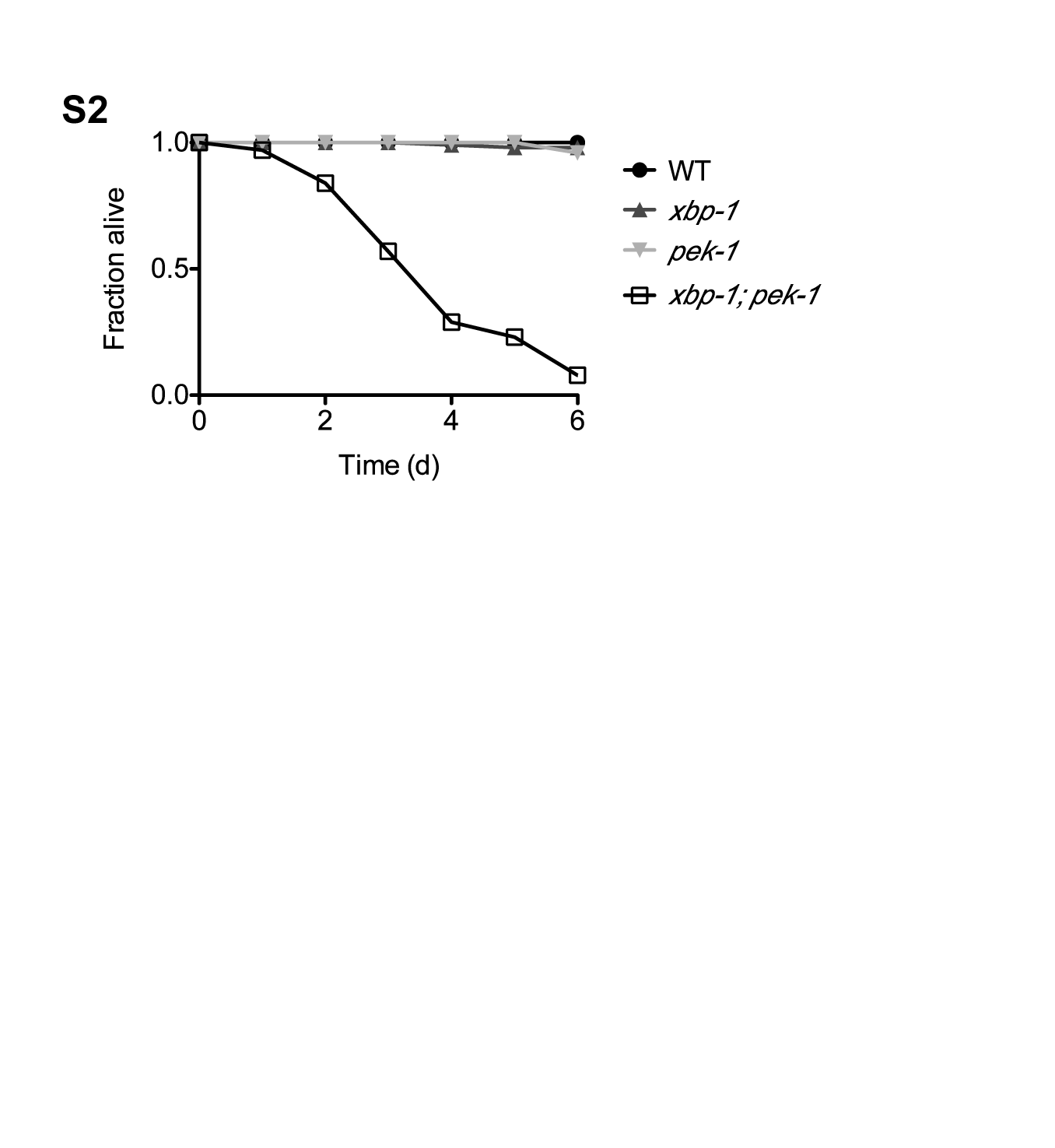

Supplement: Figure S2 — The UPR protects against lethality in the presence of pathogen during adulthood. Survival of WT, xbp-1(tm2482), pek-1(ok275), and xbp-1(tm2482); pek-1(ok275) strains grown at 16°C to the L4 stage, then shifted to plates seeded with P. aeruginosa PA14. Results are representative of two independent experiments. (TIFF) [file pgen.1002391.s002.tiff]

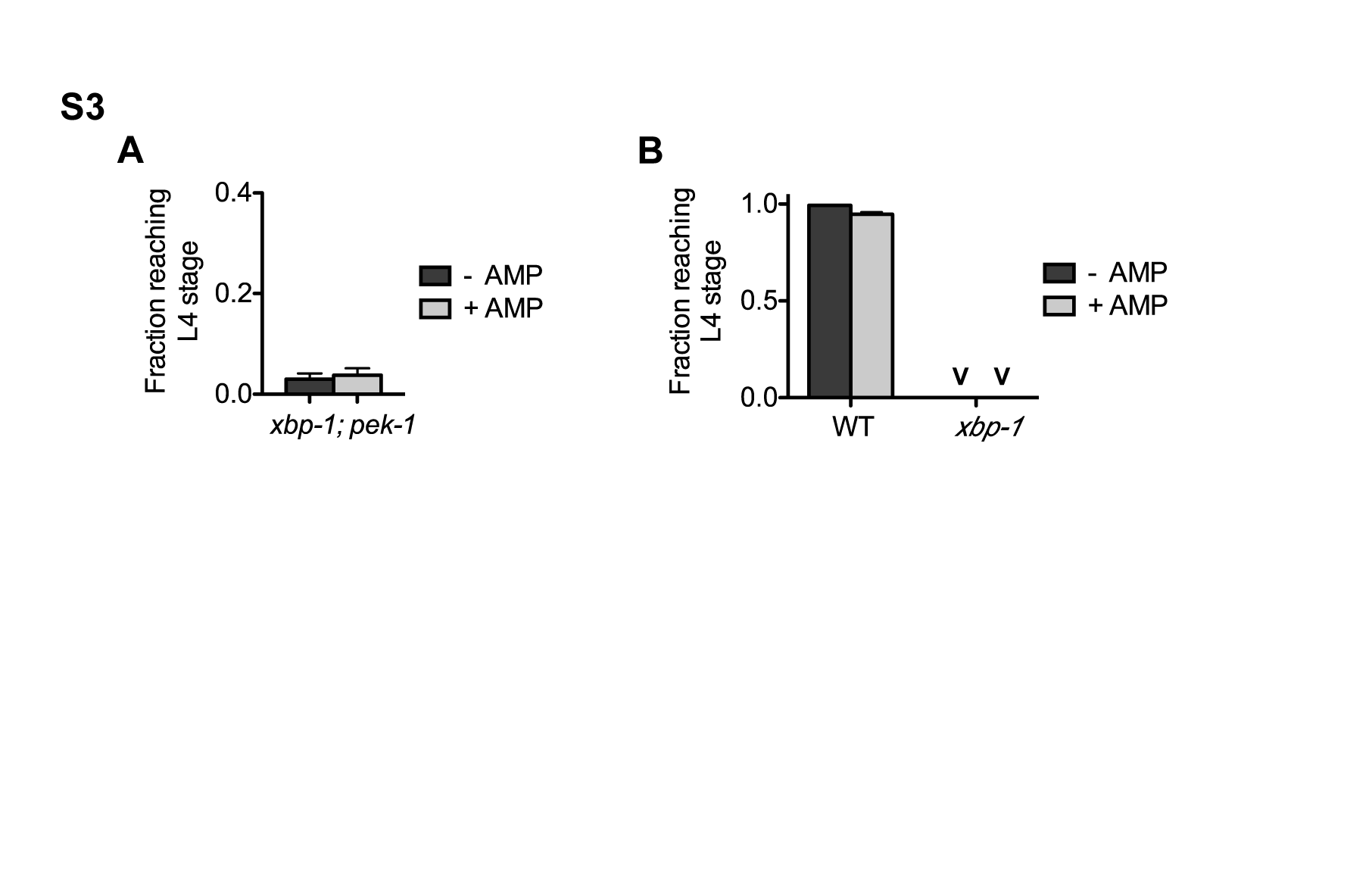

Supplement: Figure S3 — Temperature-sensitive lethality of the xbp-1; pek-1 mutant is not caused by increased pathogenicity of E. coli OP50 at elevated temperatures. (A) Development of xbp-1(tm2482); pek-1(ok275) mutant from eggs on E. coli OP50 after 3 d at 23°C with or without the bacteriostatic drug ampicillin. (B) Development of the N2 WT strain and the xbp-1(tm2482) mutant from eggs on E. coli OP50 after 3 d at 27°C with or without the bacteriostatic drug ampicillin. Values represent average fraction of eggs developed to the L4 larval stage or later ± s.e.m. (n = 2 independent experiments). (TIFF) [file pgen.1002391.s003.tiff]
